# Is multiple nest building an adequate strategy to cope with inter-species nest usurpation?

**DOI:** 10.1186/s12862-016-0671-7

**Published:** 2016-05-06

**Authors:** Petra Sumasgutner, Juan Millán, Odette Curtis, Ann Koelsag, Arjun Amar

**Affiliations:** Percy FitzPatrick Institute of African Ornithology, DST-NRF Centre of Excellence, University of Cape Town, Rondebosch, 7701 Cape Town, South Africa; Biological Sciences, University of Cape Town, Rondebosch, 7701 Cape Town, South Africa

**Keywords:** Inter-species competition, Phenotypic trait, Behavioural plasticity, Game theory, Colour polymorphism, Urbanisation, Colonisation, Raptor, Black sparrowhawk, Egyptian goose

## Abstract

**Background:**

Black sparrowhawks *(Accipiter melanoleucus)* recently colonised the Cape Peninsula, South Africa, where the species faces competition for their nest sites from Egyptian geese *(Alopochen aegyptiaca)* which frequently usurp black sparrowhawk nests. In this paper, we test the hypothesis that multiple nest building by black sparrowhawks is a strategy to cope with this competitor, based on a 14-year long term data set.

**Results:**

Two main results support the hypothesis: first, the numbers of intact nests per breeding season in black sparrowhawk territories increased as levels of geese interactions increased, specifically when usurpation occurred. Usurpation occurred significantly more often at nests later in the season, and may provide a further explanation for the advancement of the black sparrowhawk breeding season towards earlier breeding attempts which results in an overall extension of the breeding period (over 9 months) that has been found in our study population. Second, nest usurpation had a negative impact on black sparrowhawks’ reproductive performance at the ‘nest’ level, but not at the ‘territory’ level when multiple nests were available within the same breeding season, suggesting that this strategy was effective for dealing with this competitor. However, our results do not rule out long term negative consequences of these interactions, for example, reduced adult survival rates or reduced lifetime reproductive success, due to the higher energy demand required to build several nests each breeding season.

**Conclusions:**

Our results suggest that black sparrowhawks avoid direct conflict with this large and aggressive competitor and instead choose the passive strategy in allocating more resources to multiple nest building. Our research further highlights the importance of behavioural plasticity, which might be especially important for city-dwelling species in the face of global urbanisation.

**Electronic supplementary material:**

The online version of this article (doi:10.1186/s12862-016-0671-7) contains supplementary material, which is available to authorized users.

## Background

Nest structures are essential for reproduction in many bird species, which typically build one nest per breeding attempt. Some species, however, build more than one nest (or nest-like structures), sometimes prior to incubation (e.g. [1–3]). The reason for this behaviour is often unclear, especially since nest building requires considerable energy [4], not only in gathering resources to build the nest itself, but also in defending the nests against conspecific and inter-species competitors [5, 6]. In fact, to reduce the costs of nest building, some species may even reuse the same nests or nesting materials from previous nests [7, 8], or steal the nesting material from conspecifics or other species [9, 10]. For species sharing the same habitat, evolutionary niche partition may select for non-overlapping nest niches [11]. However, competition can result in nest usurpation [12, 13] if costs of building a nest are either too high, the competitors’ nest constitutes a more optimal resource [11] or usurpation occurs due to inter-species attraction and habitat copying (i.e. the presence of another species is used as a cue for a high quality habitat; see for example [14]).

Nest usurpation is relatively common among birds (see [11] reviewing usurpers in 17 avian families worldwide and species being usurped in 18 families; this includes 10 African usurping species described by Martin, Broekhuysen [15]). Inter-species nest usurpation is here defined as one species using another species’ nest as its own, in line with the definition by Lindell [11] and different from brood parasitism, where eggs are laid in another species’ nest without providing parental care [11]. Nest usurpation can be limited to the use of non-active or abandoned nests, or may involve violent takeovers. The latter is the focus of our current study.

Nest usurpation is likely to be energetically costly for the victim, both, directly, because of the associated reduction in reproductive success, and indirectly, since they will have to invest additional resources and time into building an alternative nest or will have to delay breeding until the nest is vacant again. From an evolutionary perspective it is therefore expected that different strategies have evolved to avoid nest usurpation, from conspecifics and inter-species competitors, and/or reduce negative fitness consequences. For example, some species spend considerable resources indicating territory occupancy [16], actively and aggressively defending their nest sites [17] or trying to reduce conflicts with competitors via the spatial configuration of their nests [18]. Alternatively, in cases where nest abandonment before egg-laying is relatively common, multiple nest building might be employed. For example in the grey fantail (*Rhipidura albiscapa*) multiple nests are related to an effort to conceal the ‘active’ nest from predators (i.e., multiple nests reduce the chance that the predator will find the one nest that actually contains eggs [3]). The predator’s search image for nests usually changes with nest density [19, 20], in a way that the predator expects eggs or nestlings to prey on when finding a nest, but when multiple nests are available and thus the effort in finding the active nest is increasing, the predator might give up the nest searching more quickly.

One approach proposed as an effective strategy to cope with nest usurpation specifically is through building alternative nests, either to increase the visual presence on the territory [21–23] or to provide a ready-to-use nest in case of harassment or usurpation [24, 25]. Such an approach would minimise the time required to re-initiate a breeding attempt in the event of nest usurpation. Although multiple nest building might be a costly strategy, given the energy and resources required to build several nests on territory [26], it may still constitute the most cost effective strategy in terms of preventing injuries through direct conflicts and hence increasing an individual’s long-term fitness [27, 28].

In this study, we test if black sparrowhawks (*Accipiter melanoleucus*) breeding on the Cape Peninsula use multiple nest building as a strategy to cope with Egyptian geese (*Alopochen aegyptiaca*) nest harassment and usurpation. Both species have recently expanded their South African range into the south-west and now breed in the urban and suburban habitats of Cape Town (see details in Amar et al. [29] on black sparrowhawks, and in Mangnall, Crowe [30] on Egyptian geese). Additionally, this population has extended its breeding season towards earlier laying together with an overall prolongation of the breeding period in comparison with the historical range [31].

Previous observations suggest that nest harassment or usurpation by Egyptian geese occurs in 2/3 of black sparrowhawk nest sites, and frequently results in nest failure [24]. In this same study it was also observed that multiple nest building might predominantly occur on territories where geese were present [24], although this idea was never explicitly tested. If the ‘multiple nest building strategy’ is an effective method for coping with the pressure of nest competitors, we might expect that pairs building multiple nests would maintain similar breeding performance despite being in areas with high levels of geese interactions. Further, the strategy might be especially effective if combined with early breeding, a hypothesis previously suggested by Martin et al. [31] as one explanation for the observed earlier extension of the breeding season for this range expanded population. Earlier laying by black sparrowhawks might thus be aimed at reducing nest competition with Egyptian geese by temporal separation.

Following the range expansion of the black sparrowhawk, the species now displays clinal variation in the frequencies of dark and light morph adults [32], with the Cape Peninsula population showing the highest proportion of dark morph adults (c. 75 %) within South Africa [29], whereas the light morph is numerically dominant (c. 80 %) in the rest of the species distribution. In several polymorphic species different morphs exhibit various levels of aggression, whereby melanistic individuals are usually more aggressive [33–35] than the lighter morphs, as well as less sensitive to stress [36, 37]. Thus, it might be that the different colour morphs in this black sparrowhawk population show different strategies for coping with Egyptian geese, with one morph pursuing a more passive strategy, for example multiple nest building, whilst the other morph might adopt a more active strategy, for example, defending their single nest site against usurpation.

In this study we use long-term (14-years) data from an intensively monitored population of black sparrowhawks on the Cape Peninsula to test several hypotheses relating to black sparrowhawk – Egyptian geese interactions:

(H1) Multiple nest building by black sparrowhawks is a strategy to cope with competition by Egyptian geese. If so, we would predict that black sparrowhawk territories with multiple intact nests in the same breeding season will occur when there are high levels of geese interactions.

(H2) The probability of interactions between Egyptian geese and black sparrowhawks has a temporal pattern and is not randomly distributed throughout the breeding season. Specifically, we predict that conflicts with geese may increase as the breeding season progresses. We make this prediction based on the results of Martin et al. [31], which showed that the black sparrowhawk, which recently colonised this area of South Africa had advanced its timing of breeding and speculated that this had occurred as a strategy to avoid conflicts with geese. We further predict that frequencies of geese present on territory have increased over the study period of 14 years together with the frequency of multiple-nest building, but that the frequency of nest harassment and nest usurpation has decreased.

(H3) Harassment of breeding black sparrowhawk pairs and nest usurpation have a negative impact on reproductive success at the nest level, but not at the territory level when multiple nests are available. If the multiple nest building strategy is effective, we expect an equal reproductive performance at the territory level for sites with multiple nests in the same breeding season, irrespective of the level of geese disturbances.

(H4) There are morph-specific differences in the number of nests built on a given territory, and the frequency of nest harassment and usurpation by Egyptian geese. If different morphs exhibit differential strategies for coping with this competition, we would predict that dark morphs might adopt a more active strategy in defending their single nest, and light morphs a more passive strategy in building multiple nests on their territories. Hence, we expect nest usurpation to be less frequent in dark morphs than in light morphs, but multiple nests on territory to occur more often in light morphs than in dark morphs. At the same time nest harassment might be higher in dark morphs than in light morphs, if dark morphs are indeed defending their nest more actively against competitors, while light morphs are deserting earlier.

## Methods

### Study area

The Cape Peninsula is about 470 km^2^ in size and forms the south-western end of the Cape floristic region making it an important biodiversity hotspot [38]. The habitats found on the Peninsula range from native Fynbos shrubland to both indigenous Afromontane forest and exotic *Pinus* and *Eucalyptus* plantations [39], wetlands and several artificial habitats like gardens, golf courses, vineyards and highly urbanised areas (sealed soil covered by buildings and traffic structures). The Cape Peninsula experiences a Mediterranean climate and receives winter rainfall (mean annual rainfall: 1250 mm; average monthly temperatures: 12–21 °C [38]). The monitored black sparrowhawk territories (estimated home ranges captured in a 3x3km buffer; see Sumasgutner et al. [40] for details on the method) consists of 39.8 % open landscapes, 25.4 % forest, 23.3 % urban areas, 5.1 % vineyards, 3.7 % gardens and 2.7 % wetlands and water bodies.

### Black sparrowhawk long-term monitoring

The colonisation by the black sparrowhawk (♂ 430–490 g, ♀ 650–980 g; [41]) of the Cape Peninsula, Western Cape, was first recorded in the 1990s [42, 43]. The breeding population in Cape Town has been monitored since 2000, when there were three occupied territories monitored, and has increased to around 50 pairs monitored over the last 14 years [31], with a density of 17.6 territories/100 km^2^ (recorded 2013). The population is expanding; hence the higher number of occupied territories is not the result of a higher search effort. The first three territories in 2000 were located in an area of only 0.9 km^2^, whereas the 54 territories in 2012 were distributed within 314 km^2^. Occupied territories were located by surveying suitable stands of trees during the breeding season, searching for calling sparrowhawks, prey remains, whitewash and nest structures. Territories were classified as active if birds were seen on territory and at least one nest appeared as built on or was decorated with fresh greenery during the breeding season between March (Austral early autumn) and October (spring). In this study we only included intact nests in active territories, and excluded territories classified as vacant, with no black sparrowhawks observed on territory over the season. The term multiple nests refers to the number of intact nests per active territory per breeding season. Because of the small sample size of territories with more than three intact nests we used the following ordinal categories in our analyses: 1 = only one single nest on territory, 2 = two nests in separate trees within the same territory, and 3 = three to six nests in separate trees within the same territory per breeding season.

Black sparrowhawks on the Cape Peninsula usually build one or several new nests each season rather than having just one traditional nest that they reuse each breeding season (personal observations of the authors), similar to other *Accipiter* species (for example the Eurasian sparrowhawk *A. nisus*, [44]). Nest reuse might also be rare because previous nests are most likely destroyed by winds between breeding seasons. Usually multiple nests were built on the nest debris of previous sites and freshly decorated with greenery, which we often observed at the same time as a new nest was initiated, or several new nests were initiated sequential during the courtship period, or another nest was initiated soon after incubation failed at an early stage. Territories were visited regularly (approximately monthly) throughout the breeding season until a breeding attempt was detected. As soon as territorial birds were observed in courtship (mating heard or seen) or incubation behaviour (female sitting low on nest) monitoring increased in frequency (approximately weekly) to check on the progress and success of each breeding attempt (see [45] for more details). Most breeding birds in our population are individually colour ringed (see [29]) and have a high partner and site fidelity [45]. When chicks were between 3–5 weeks old they were fitted with a uniquely coded SAFRING band and an individualised combination of two metal colour rings. The number of chicks on the nest at this stage was used as our measurement of productivity, but nest success was again confirmed at the time of fledging, to ensure that no brood failure occurred later on. Two measurements of productivity were used in our study: ‘productivity per breeding attempt’ (on nest level, 0–3 chicks) and ‘annual territory productivity’ (0–4 chicks per territory and breeding season, which goes up to a max of 4 due to occasional successful double brooding [39]).

We expressed timing of breeding in two different ways: Firstly, by dividing the breeding season into two periods: ‘early’ (egg-laying March to May) and ‘late’ (egg-laying June to October). This division was previously used by Martin et al. [31] and was based on the observed timing of breeding in the species ‘historical’ range where >95 % of breeding attempts occurred in the ‘late’ breeding period. Since Martin et al. [45] suggested the temporal separation from the peak breeding season of Egyptian geese as one mechanism that has potentially driven this extension of the breeding period towards earlier egg-laying, we used the exact same (timing of breeding) parameter to test this hypothesis. Secondly, we also treated the month of egg-laying as an ordinal variable called ‘lay month’, where month was assigned a rank between 3 = March and 10 = October, this was treated as a continuous variable in our analyses. This provides a higher resolution analysis of the timing of breeding and was further used as a co-variable in productivity analyses.

### Egyptian geese *(Alopochen aegyptiaca)*

Egyptian geese (1500–2348 g [47]) are widely distributed in Africa and breed north and south of the Sahara and throughout the year, mainly in winter between May and October with a peak in August and September [46, 47]. In South Africa, Egyptian geese numbers have increased with urbanisation and agriculture [48]). Egyptian geese are known to be territorial [49], but are not capable of building their own nests; they breed on the ground but also in trees where they use both, vacant nests of other species, but also usurped nests after violent takeovers [50]. Geese have been recorded on other raptors’ nests [49] including breeding attempts on Verreaux Eagle (*Aquila verreauxii*) nests. Egyptian geese are precocial birds and use these nests for a relatively short period of time (28–30 days of incubation), then hatchlings and adults leave the nest [51]. In the Western Cape, numbers of Egyptian geese are increasing rapidly since the 1980s, a phenomenon specifically reported for urban areas [24, 52]. In our study area, Egyptian geese sometimes use nests after black sparrowhawks have finished nesting. However, we do not include such data in our analyses because these were occasional observations and not systematic records. The weekly monitoring of black sparrowhawk territories during breeding decreases in frequency to an approximate monthly visit as soon as juveniles fledge to capture the rare event of double broods [39]. For the same reason we have no knowledge if the breeding attempts by Egyptian geese in the usurped nests are successful (i.e., chicks hatched), nor how many chicks they might produce.

### Conflict with Egyptian geese

For each intact black sparrowhawk nest we provided a set of binary scores (1 or 0), as follows: (i) firstly, if Egyptian geese were present during any monitoring visit over the entire season or not (1 = geese present, 0 = no geese ever present), (ii) secondly, if geese harassment was ever seen during the breeding season or not (i.e., a goose was seen at least once directly at the nest) and (iii) thirdly, if geese usurpation occurred or not (i.e., a goose had taken over the nest and laid its own eggs).

All our geese-scores were highly correlated and thus we either used them separately or we used a *‘nest g-score’* on the nest level by adding together the binary scores. This latter value ranged from 0 = no geese present, 1 = geese present, 2 = nest harassment to 3 = nest usurpation. When considering this measure, it is important to note that nest usurpation does not necessarily result in nest failure by the black sparrowhawk because this is highly dependent on the timing of usurpation. During the pre-laying period or early stages of incubation with immediate re-laying (in the same nest) it might not cause nest failure if the black sparrowhawk pair manages to win the nest back in a short period of time instead of abandoning it immediately.

These data (based at the nest level) were also used to give a ‘*territory g-score*’ as the minimum value factor at a territory level ranging from 0 = no geese ever seen on territory, 1 = geese seen on territory, 2 = at least one nest harassment witnessed and 3 = at least one nest usurped by geese. The territory g-score therefore reflects the maximum score of any single nest on territory on a yearly basis. This territory g-score was then used in our analysis to test the influence of Egyptian geese on multiple nests within territories for each breeding season and on the black sparrowhawks’ breeding parameters.

### Statistical analysis

The R packages ‘lme4’ [53] and ‘MuMIn’ [54] were used to implement generalised linear mixed models. Nest ID (for models on nest level), territory ID (for models on nest and territory level) and study year were included as random factors to control for pseudo-replication [55]. Nest and territory controlled for repeated broods fledged from the same location, i.e. the same nest or the same territory, and year controlled for broods fledged in the same year. All covariates were tested beforehand for correlation of fixed effects (Spearman’s rank correlation). Error family was chosen according to the type of response variable: binomial error family and logit link function for geese present, nest harassment and nest usurpation and poisson error family and log link function for the number of nests on territory. For productivity (number of fledged) we used the package ‘nlme’ [56] to call the quasi poisson family and control for overdispersion in our data. The models included our ‘*nest g-score’* or our ‘*territory g-score’* as an index for geese interactions, together with the male and female morph and their interaction as fixed effects. Post-hoc comparisons between factor variables were performed using the package ‘lsmeans’ [57]. Residual distributions of the models were inspected to assess model fit. All statistical analyses were performed with the software R version 3.1.3 (2015–03–09, R Development Core Team 2015).

## Results

The first territory with more than one intact nest in a breeding season was detected in 2002. In all analyses the data-base used covers 14 years of monitoring (2000–2013), with 412 separate breeding attempts recorded on 71 different territories (i.e., locations and breeding pairs). Of these breeding attempts 276 broods were successful (67 % with at least one fledgling) and 136 broods failed (33 %). 32 of the 71 territories (45 %) had more than one intact nest in at least one breeding season, and in these territories, when multiple nests occurred, they ranged between two and six nests (14 territories had up to three intact nests per breeding season, and respectively one had four and one had six nests). At 64 territories (90 %) geese were present in at least one breeding season; we recorded geese harassment at 53 territories (75 %) and nest usurpation at 43 territories (61 %).

### Multiple nests on territory

**Fig. 1:**
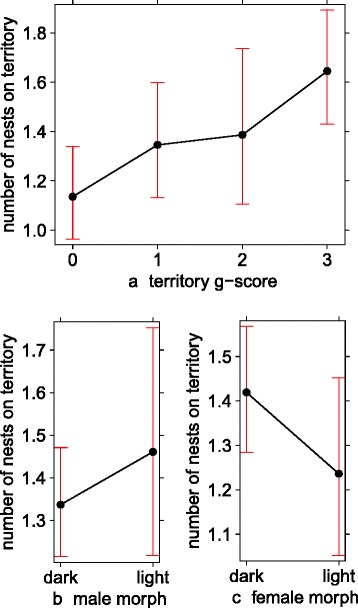
Number of intact black sparrowhawk nests per active territory (71 territories in 14 years; *N* = 396 records) in each breeding season in relation to (**a**) the territory g-score (0 = no geese present, 1 = geese present, 2 = nest harassment and 3 = nest usurpation), (**b**) the morph of the breeding male and (**c**) the morph of the breeding female. Figures based on predicted values of GLMMs, error bars represent 95 % CIs

The full model included the number of nests (on territory in each breeding season) as response variable and the territory g-score, male morph and female morph including their interaction as explanatory variables (together with territory ID and study year as random terms). We found that higher levels of geese interactions, in terms of our territory g-score, correlated with a higher number of intact nests within black sparrowhawk territories (GLMM, ‘territory g-score’ term: *χ*^*2*^_*(df=3)*_ = 11.42, *P* = 0.009, *N* = 396 territories; Fig. 1a). Post-hoc pairwise comparisons revealed a significant difference in the numbers of nests present on territories where no geese were present and territories that experienced nest usurpation (territory g-score 0 and 3 comparison; estimate 0.37 ± 0.11 SE, *z*-value = 3.35, *P* = 0.004). All other comparisons between different levels of the territory g-score were not significant. We found no support for morph differences of either sex in the number of nests on territory (‘male morph’ term: *χ*^*2*^_*(df=1)*_ = 0.19, *P* = 0.67, Fig. 1b; ‘female morph’ term: *χ*^*2*^_*(df=1)*_ = 2.37, *P* = 0.12, Fig. 1c) nor support for morph differences when using the combination of male and female morph and the number of nests on territory (‘morph interaction’ term: *χ*^*2*^_*(df=1)*_ = 0.35, *P* = 0.55).

### Temporal variation in goose conflict over the black sparrowhawk’s breeding season

**Fig. 2:**
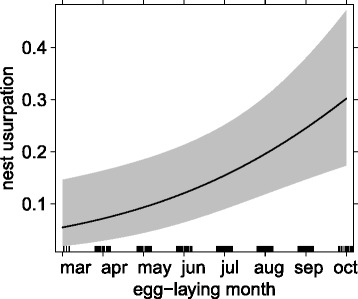
Probability of Egyptian geese usurping black sparrowhawk nests dependent on the timing of breeding (egg-laying between March = 3 and October = 10 ; *N* = 358 nests). Figure based on predicted values of GLMMs, 95 % CIs in shaded grey; black bars on the x-axis represent sample size

We ran three full models separately including (i) geese presence, (ii) nest harassment or (iii) nest usurpation as binary response variables with breeding period (early *versus* late), male morph and female morph including their interaction as explanatory variables (together with nest ID and study year as random terms). At the nest level, we found no temporal variation in geese presence or nest harassment (*N* = 358 nests; GLMM with geese presence as response: ‘breeding time' term: *χ*^*2*^_*(df=1)*_ = 1.57, *P* = 0.21; GLMM with nest harassment: ‘breeding time' term: *χ*^*2*^_*(df=1)*_ = 0.71, *P* = 0.41), but the probability of nest usurpation was significantly higher in the late breeding period than in the early breeding period (GLMM, ‘breeding time’ term: *χ*^*2*^_*(df=1)*_ = 4.58, *P* = 0.032, *N* = 358 nests). We fitted an alternative model using the month of egg-laying as an explanatory variable instead of pooling the breeding time in two periods as previously used by Martin et al. [31], and obtained a stronger result (GLMM, ‘egg-laying month’ term: *χ*^*2*^_*(df=1)*_ = 8.84, *P =* 0.003, *N* = 358 nests; Fig. 2).We again found no support for morph differences (sex-morph in isolation or in their interaction) in the probability of geese present on territory, nest harassment or nest usurpation.

### Temporal variation in the goose conflict over the study period

When using ‘year’ as fixed continuous effect to explore for a trend in goose conflict over the study period, we found the probability of ‘geese present’ on territory slightly increasing over time (*χ*^*2*^ = 2.25, *P =* 0.13), but nest harassment decreasing (*χ*^*2*^ = 3.37, *P =* 0.066) as well as nest usurpation significantly decreasing over the 14-year study period (*χ*^*2*^ = −0.47, *P =* 0.002). We found no indication for a year effect on the number of nests on territory (GLMM ‘year’ term: *χ*^*2*^_*(df=1)*_ < 0.01, *P =* 0.97, *N* = 396 territories).

### Multiple nest building by black sparrowhawks as a strategy to cope with Egyptian geese

**Fig. 3:**
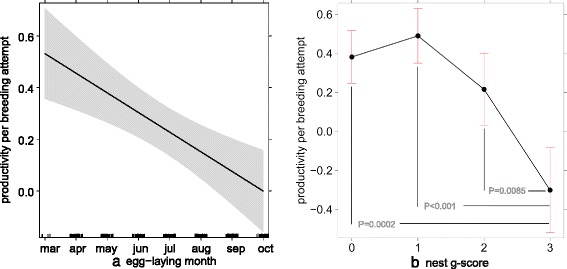
Nest level productivity (0–3 chicks per breeding attempt, *N* = 380 nests) depending on (**a**) the month of egg-laying (black bars on x-axis represent sample sizes); and, (**b**) geese interactions on nest level: no geese present, geese present, nest harassment and nest usurpation (‘nest g-score’ ranging from 0–3). P-values indicate significance in post-hoc comparisons. Figure based on predicted values of GLMMs, 95 % CIs in shaded grey or error bars; model details are given in Table 1

Nest success was highly dependent on the breeding time (month of egg-laying) and geese interactions on nest level (variable ‘nest g-score’, *χ*^*2*^_*(df=3)*_ = 41.19, *P* < 0.001). Although this result is influenced mainly by nest usurpation (see model details Table 1, and post-hoc comparisons in Fig. 3a-b).

**Table 1:** Parameter estimates of the traits explaining variation in nest level productivity (0–3 chicks per breeding attempt, *N* = 380 nests), controlling for the month of egg-laying. GLMM fitted with quasi poisson family and log-link function

Fixed effects:	Parameter estimate ± SE	*t*- value	*Pr(>|t|)*
Egg-laying month	–0.08 ± 0.02	–3.73	0.007
Nest g-score: Geese present ^a^(*n =* 283)	0.11 ± 0.09	1.17	0.282
Nest harassment (*n =* 172)	–0.17 ± 0.11	–1.49	0.180
Nest usurpation (*n =* 102)	–0.68 ± 0.13	–5.35	0.001
(Intercept)	0.92 ± 0.15	6.17	<0.001

^a^No geese present was the reference category

**Fig. 4:**
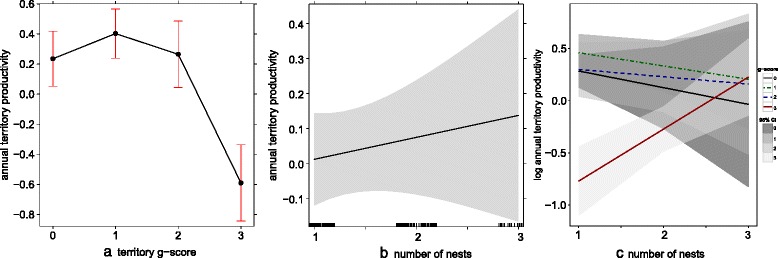
Annual territory productivity (0–4 chicks per season, *N* = 433 territories) depending on (**a**) geese interactions on territory level: no geese present, geese present, nest harassment and nest usurpation (‘territory g-score’ ranging from 0–3); (**b**) the number of nests on territory (where 3 = 3 or more nests; black bars on x-axis represent sample sizes); and, (**c**) the interaction term between the g-score and the number of nests on territory. Figure based on predicted values of GLMMs, 95 % CIs in shaded grey or error bars; model details are shown in Table 2

Annual territory productivity was highly dependent on the territory g-score (Fig. 4a), specifically in interaction with the number of nests on territory (Fig. 4b) whereby productivity was unrelated to the number of nests for the lower g-scores (0 = no geese, 1 = geese present, 2 = nest harassment), but showed a significantly positive relationship with the number of nests on territories experiencing nest usurpation (Fig. 4c, red line, model details in Table 2). This result was principally driven by single nest territories that experienced usurpation which had considerably lower productivity than multiple-nest territories (i.e. ≥3 nests; Fig. 4c).

**Table 2:** Annual territory level productivity (0–4 chicks per season, *N* = 433 territories) depending on the number of nests on territory, the territory g-score and the interaction between these two terms. GLMM fitted with quasi poisson family and log-link function

Fixed effects:	*χ2*	Df	*Pr(>χ2)*
Number of nests	0.49	1	0.482
Territory g-score	34.30	3	<0.001
Interaction: no. of nests*g-score	14.47	3	0.002
(Intercept)	3.29	1	0.070

*indicating an interaction term between fitted fixed-effects

## Discussion

Three main results emerged from our study and provide support for several of our hypotheses. Firstly, black sparrowhawks build more nests in their territories when they experience a high level of geese interactions. This result provides support for our first hypothesis (H1), that multiple nest building by this raptor species might help in coping with nest site competition from geese. This result was not driven by the presence of Egyptian geese on territory or by geese harassing black sparrowhawks on nests, but, specifically, by nest usurpation. Only on territories experiencing usurpation were numbers of nests higher than on territories where no usurpation was recorded. Secondly, we found that geese interactions, specifically nest usurpation, had a negative impact on the black sparrowhawks’ reproductive performance at the nest level, but at the territory level, multiple nests eliminated this negative impact, thus providing support for our third hypothesis (H3) that multiple nest building is an effective strategy for coping with nest competition from Egyptian geese. Thirdly, we found that usurpation was more common on black sparrowhawk nests initiated later in the season, thus providing some support for our second hypothesis (H2), that the longer breeding period, specifically the extension of the season towards earlier breeding attempts in this population (as described by Martin et al. [31]) could also be driven by competition with geese. Finally, we found no indication that dark morphs were more frequently harassed or less frequently usurped by geese than light morphs, or that dark morphs less frequently built multiple nests than light morphs, thus providing no evidence for our last hypothesis (H4) that different morphs adopt different strategies to cope with Egyptian geese.

The aggressiveness of Egyptian geese when attacking black sparrowhawk nests in our population has been documented with camera footage [58]. Geese harassment can be violent, intense, and so frequent that it can result in nest desertion and brood failure [24]. Our monitoring data include a report of an adult black sparrowhawk male being killed by a goose [59]. It is therefore not surprising that strategies have evolved to cope with the threat of nest usurpation. Following Smith’s (1982) ‘Hawk vs. Dove Game Theory’: when two species of different size and strength are competing for the same resource, the bigger, stronger one is assigned the role of ‘Hawk’ (the Egyptian goose) while the smaller, weaker one is assigned the role of ‘Dove’ (the black sparrowhawk). When ‘Doves’ are forced to compete with ‘Hawks’ for a certain resource, a nest for instance, the ‘Dove’ will relinquish the resource before being harmed. Hence, in this manner the ‘Dove’s’ overall fitness will be less affected than by confronting the ‘Hawk’ directly and risking severe injuries or even death. If this Game Theory is applied to the black sparrowhawk and Egyptian geese conflict, the costs, in terms of energy needed to build multiple nests for sparrowhawks might, overall, be smaller than the costs in terms of both the energy needed to actively confront geese and the risk of injury. Additionally, in assessing the costs and benefits of the different strategies, it must also be considered that black sparrowhawks (an altricial species) need a nest over several months (38 days of incubation, and a further 37–50+ days until fledging [39, 60]), to complete the breeding cycle, while geese require only 28–30 days for incubation [51]. This difference in the time of nest-usage might further favour nest desertion over violent confrontation. Also, black sparrowhawks on the Cape Peninsula experience a long breeding season, lasting over 9 month. A delay in egg-laying might therefore not have such a high impact as has been documented for birds in northern environments (e.g., [61–63]). Thus, the passive strategy of multiple nest building might be limited to systems with an protracted breeding season, e.g. in tropical environments. For example in Mariana crows (*Corvus kubaryi*) multiple nest building is very common, with frequent renesting attemps after previous nests failed and occasional double brooding [64]. Successful double brooding is also known for the black sparrowhawk (11 double breeding attempts between 2000 and 2013, 6 of which were successful; see also [39]).

In this study we focused on multiple nest buidling as a strategy prior to incubation. However, our records might include replacement nests since there is a possibility that completed but empty nests found in the study area might have failed very early during egg-laying (as for example shown by Maddox, Weatherhead [65] for cryptic predation) which is difficult to detect, rather than being abandoned. This potential early nest failure is especially difficult to quantify in a raptor that nest high up in trees in dense forest areas and the demands of frequently monitoring up to 54 sites. One indication that some of the multiple nests might indeed have recieved eggs and are in fact replacement nests after early brood failure is that only nest ursurpation by Egpytian geese, but not their harassment or their sheer presence on territory predicted multiple nest building.

Multiple nest building is known for many raptor species, with multiple nests build over many years, but also within one season [21, 23]; our results are in line with the ‘competition avoidance by nest site hypothesis’ discussed for both, within species competition (see [66] and citations therein) and inter-species competition ( e.g., in golden eagles *Aquila chrysaetos*, Bonelli's eagle *Hieraaetus fasciatus* [67] and ospreys *Pandion haliaetus*) [68]. Raptors should derive some evolutionary advantage from this behaviour since natural selection would be expected to favour behaviours that tend to economise effort. On the other hand sexual selection (e.g., if multiple nests are used to advertise the quality of the male) could favour behaviours that actually do not economise effort, as for example shown in many passerines [2, 69–72]. Beside competition avoidance, there are several other non-exclusive purposes which might explain multiple nest building in raptors: (i) nests as an advertising signal of territory-ownership [22, 23], (ii) reduction of nest ectoparasites, or (iii) ‘frustration nests’ following a reproductive failure [73]. All these proposed mechanisms illustrate that this behaviour is a common life history trait in many different raptor species – it is therefore not surprising that black sparrowhawks make use of multiple nest building to cope with the competition posed by Egyptian geese if needed. It appears that pairs that are not usurped by Egyptian geese carry on with their traditional, single nesting behaviour, but when goose usurpation occurs they may adapt the multiple nest building strategy. This was tested in *hypothesis 1*; where our results showed that nest usurpation by geese was related to the number of black sparrowhawk nests built (Fig. 1a).

Coincidental with their range expansion, the black sparrowhawks on the Cape Peninsula prolonged their breeding period towards earlier breeding attempts [31]. Egg-laying in this regions occurred between March and October, with earlier breeding attempts having significantly higher breeding success than later attempts (Table 1 and Fig. 3a). We tested in *hypothesis 2* whether this phenomenon may be linked to geese usurpation (and corresponding nest failure), since we found that usurpation increased significantly later in the season (Fig. 2). Egyptian geese in South Africa breed all year round, with a peak between August and September; thus, the advancement in laying by black sparrowhawks may be aimed at reducing nest competition by temporal separation. Martin et al. [31] also tested the hypothesis that the timing of breeding and productivity are linked to local weather conditions, but found no evidence that breeding parameters are affected by rainfall or temperature. While climatic variation can have important implications for populations, other aspects of environmental change, as for example inter-species interactions might have a stronger influence on the breeding phenology, specifically in rapidly developing urban areas. However, range expansion of one or both species in this study might have been facilitated by climate change, as was recently shown in pied crows (*Corvus albus*) in this region [74]. In a similar manner the morph distribution of black sparrowhawks might be connected to changes in light levels across South Africa [75]; and solar radiation will likely be affected by global warming.

Alternatively the number of nests might also reflect the quality of a territory in regard to prey availability (i.e., multiple-nest building might occur more frequently in high quality habitats because only then the energetic costs of establishing several nest might be overridden by the benefits of nesting in a high quality territory). However, a recent short-term study on the diet of black sparrowhawks did not show any temporal variation in diet composition or prey abundance within our study area [76] making food availability an unlikely mechanism to explain the timing of breeding or variation in productivity in black sparrowhawks.

Most importantly we found strong support for *hypothesis 3*, that the strategy of multiple nest building appears to be a successful one. As predicted, productivity decreases with geese usurpation at the nest level, but is clearly maintained high at the level of the territory when multiple ready to use nests are available. There is also a slight productivity decrease with g-scores 0 (no geese present) to 2 (nest harassment), that could in theory reflect the costs of multiple nest building in absence of nest usurpation. We have no data on the actual costs of nest building in black sparrowhawks, but it is supposedly energy demanding [4]. There is an apparent increase in the slope from g-score 0 (most negative) to g-score 3 (clear positive) which could indicate that the cost of multiple nest building becomes overridden by the benefits as geese interactions become more severe (Fig. 4c).

We found no support for *hypothesis 4*, that dark and light morph black sparrowhawks have adopted different strategies, potentially due to differences in their aggressiveness. A higher aggressiveness of dark morphs was, for instance, shown in common buzzards *Buteo buteo* [35]. However, recent studies in our population have shown that dark morphs are breeding earlier than light morphs [40], which might be an indication for their dominance over light morphs in occupying higher quality territories. There appear to be several benefits derived from earlier breeding: early broods have higher breeding success [31] and earlier nests are less frequently usurped by Egyptian geese (this study).

## Future directions

Field data on Egyptian geese would help to understand how successful their breeding attempts are early or late in the year, as well as in comparison between different nests types. This would specifically shed light on the question if nests of black sparrowhawks might be a favoured resource with specific benefits for the usurper. An extension of the study area, into more rural habitats and across South Africa, could provide new insights on the frequency and effectiveness of the multiple nest building strategy in different habitat types. Lastly, to score ‘aggressiveness’ in back sparrowhawks and disentangle the question of whether dark morphs are indeed more aggressive than light morphs, could help in relating morph-differences to the conflict with Egyptian geese.

## Conclusions

The conflict between black sparrowhawks and Egyptian geese might not only be related to the opportunistic nest use by geese but also be rooted in a conflict generated by habitat fragmentation and the overlap of nesting niches between two species adapting to the urban habitat [77]. Our results suggest that multiple nest building is indeed an efficient strategy to cope with geese usurpation and allow productivity to be maintained. To which extend this behaviour is limited to the urban habitat is currently unknown. This underlines the importance of understanding the impact of anthropogenic change on multi-species networks (e.g., reviewed in Ibáñez-Álamo et al. [78] for nest predation) and the role of behavioural plasticity. If the energy required for fighting geese (the aggressive strategy) is higher than the energy demand of building multiple nests (the passive strategy), the latter might be in the individual black sparrowhawks’ best long-term advantage. However, a potential negative impact on adult survival and/or lifetime reproductive success is yet unknown, and might affect the population viability in the long-term. In conclusion our results show that multiple nest building is most likely related to the conflict with Egyptian geese, with the decision to abandon being mediated by the risk of nest usurpation.

### Ethics

All sampling was conducted in strict accordance with current South African law and followed the Weatherall Report and the guidelines for the treatment of animals in behavioural research and teaching [79]. The protocol used for individual ringing of black sparrowhawks was approved by the University of Cape Town’s Science Faculty Animal Ethics Committee (Permit number: 2012/V37/AA).

### Consent to publish

Not applicable.

### Availability of data and materials

Morphological and ringing data on black sparrowhawks have been provided to the South African Ringing Scheme (SAFRING). All supporting data are available as supplementary material enclosed to this publication (Additional file 1).

## Additional file

Additional file 1: Supplementary material. (XLSX 64 kb)

## Abbreviations

GLMM: generalised linear mixed model
g-score: geese-score: on ‘nest’ or ‘territory’ level (0 = no geese present, 1 = geese present, 2 = nest harassment, 3 = nest usurpation)
